# Effects of high-fat diet and/or body weight on mammary tumor leptin and apoptosis signaling pathways in MMTV-TGF-α mice

**DOI:** 10.1186/bcr1840

**Published:** 2007-12-27

**Authors:** Soner Dogan, Xin Hu, Yan Zhang, Nita J Maihle, Joseph P Grande, Margot P Cleary

**Affiliations:** 1 Hormel Institute, University of Minnesota, 801 16th Avenue NE, Austin, MN 55912, USA; 2 Biostatistics Core, University of Minnesota Cancer Center, B484-4 Mayo, 420 Delaware Street SE, Minneapolis, MN 55455, USA; 3 Department of Obstetrics and Gynecology, Yale University Medical School, 300 George Street, Suite 8100, New Haven, CT 06511, USA; 4 Department of Pathology and Laboratory Medicine, Mayo Foundation, 200 Second Street SW, Rochester, MN 55905, USA

## Abstract

**Introduction:**

Obesity is a risk factor for postmenopausal breast cancer and is associated with shortened mammary tumor (MT) latency in MMTV-TGF-α mice with dietary-induced obesity. One link between obesity and breast cancer is the adipokine, leptin. Here, the focus is on diet-induced obesity and MT and mammary fat pad (MFP) leptin and apoptotic signaling proteins.

**Methods:**

MMTV-TGF-α mice were fed low-fat or high-fat diets from 10 to 85 weeks of age. High-Fat mice were divided into Obesity-Prone and Obesity-Resistant groups based on final body weights. Mice were followed to assess MT development and obtain serum, MFP, and MT.

**Results:**

Incidence of palpable MTs was significantly different: Obesity-Prone > Obesity-Resistant > Low-Fat. Serum leptin was significantly higher in Obesity-Prone compared with Obesity-Resistant and Low-Fat mice. Low-Fat mice had higher MFP and MT ObRb (leptin receptor) protein and Jak2 (Janus kinase 2) protein and mRNA levels in comparison with High-Fat mice regardless of body weight. Leptin (mRNA) and pSTAT3 (phosphorylated signal transducer and activator of transcription 3) (mRNA and protein) also were higher in MTs from Low-Fat versus High-Fat mice. Expression of MT and MFP pro-apoptotic proteins was higher in Low-Fat versus High-Fat mice.

**Conclusion:**

These results confirm a connection between body weight and MT development and between body weight and serum leptin levels. However, diet impacts MT and MFP leptin and apoptosis signaling proteins independently of body weight.

## Introduction

Breast cancer is the most common malignancy for women worldwide, with more than one million women diagnosed each year [1-3]. Although genetic mutations increase the risk for development of breast cancer, this impacts only a small percentage of women. Reproductive and lifestyle factors increasingly are implicated to play a role in breast cancer development [4]. Obesity is one of the lifestyle factors thought to affect the risk for postmenopausal breast cancer [5] and is associated with increased mortality in women diagnosed with breast cancer [6,7]. Obesity, in turn, impacts levels of serum estrogen, insulin, and/or insulin-like growth factor-1, which have all been implicated as possible links between obesity and breast cancer [8-12]. Leptin, a protein secreted primarily from adipose tissue [13-16], also has been implicated as a mammary tumor (MT) growth factor. For example, increased cell proliferation was observed when human breast cancer cells were incubated with leptin in *in vitro *studies [17-19]. Additionally, the leptin receptor was identified in breast cancer cell lines and in human breast cancer tissues [17-19].

Animal models are frequently used to gain insights into human disease processes. In general, higher body weights and/or obesity has been associated with shortened MT latency and/or increased incidence for development of spontaneous and carcinogen-induced MT in animals [20-25]. An exception noted was that genetically obese *Lep*^*ob*^*Lep*^*ob *^mice were reported to have a decreased incidence of spontaneous MTs [26], and recently our laboratory found that when *Lep *strain mice were crossbred with transgenic MMTV-TGF-α mice, obese MMTV-TGF-α-*Lep*^*ob*^*Lep*^*ob *^mice did not develop oncogene-induced MTs, although their lean littermates did [27]. The metabolic defect in *Lep*^*ob*^*Lep*^*ob *^mice is an absence of leptin [28]. In a second study, *Lepr *mice that exhibit a mutation in the leptin receptor were crossbred with MMTV-TGF-α mice and again no MTs were detected in obese MMTV-TGF-α-*Lepr*^*db*^*Lepr*^*db *^mice [29]. One could argue that MMTV-TGF-α MTs are not affected by obesity; but in an additional experiment, MMTV-TGF-α mice with diet-induced obesity had shortened MT latency [30]. Thus, in the presence of a functioning leptin axis, MTs develop. These findings led us to hypothesize that leptin is a growth factor for breast/mammary cancer cells.

In the present study, we used a dietary-induced obesity protocol to further explore the relationship of serum leptin levels and body weight with respect to MT development in MMTV-TGF-α mice. Leptin signaling proteins in MT and mammary fat pad (MFP) tissues were characterized. Although six different leptin receptor subtypes have been identified in numerous tissues and cell types, primarily the long isoform (ObRb) is responsible for activating leptin signaling pathways [31,32]. In this study, we determined expression of the total leptin receptor (ObR) as well as ObRb. Leptin acts through several signaling pathways, including Jak2/STAT3 (Janus kinase 2/signal transducer and activator of transcription 3) and PI3K/Akt [33-36], and here we determined protein and/or mRNA expression levels for these pathways. Another metabolic action of leptin that has been reported is inhibition of apoptosis in human colon [37] and prostate [38] cancer cell lines and in leukemic cells [39]. Thus, proteins involved in apoptosis (that is, total poly [ADP-ribose]polymerase [PARP], PARP [89 kDa], PARP [24 kDa], Bax, Bcl-2, and Bcl-xL) as well as caspase-3 activity were determined in MT and MFP tissues in the present study.

## Materials and methods

### Materials

Primary antibodies against Ob, ObR, phosphorylated STAT3 (pSTAT3), Jak2, phosphorylated Akt (pAkt), Bcl-2, Bcl-xL, Bax, and alkaline phosphatase-conjugated goat anti-mouse immunoglobulin G (IgG) were purchased from Santa Cruz Biotechnology, Inc. (Santa Cruz, CA, USA). ObRb was purchased from Linco Research, Inc. (now part of Millipore Corporation, Billerica, MA, USA). PARP (#9542) and anti-rabbit IgG conjugated with alkaline phosphatase were purchased from Cell Signaling Technology, Inc. (Danvers, MA, USA). β-Actin was purchased from Delta Biolabs, LLC (Gilroy, CA, USA). Enhanced chemifluorescence (ECF) substrate was obtained from Amersham Biosciences (now part of GE Healthcare, Little Chalfont, Buckinghamshire, UK). Tris-base solution, Tris/Glycine/SDS buffer, and polyacrylamide gradient gels were purchased from Bio-Rad Laboratories, Inc. (Hercules, CA, USA). Reverse transcriptase (Superscript II) and oligo(dT) were purchased from GibcoBRL (now part of Invitrogen Corporation, Carlsbad, CA, USA). Polyvinylidene difluoride (PVDF) membranes (Immobilon-P) were from Millipore Corporation. Immunoglobulin-alcaline phosphatase-conjugated secondary antibodies were purchased from Amersham Life Science Inc. (now part of GE Healthcare).

### Animals and study design

MMTV-TGF-α (C57BL6) female mice were produced and genotyped at the Hormel Institute (Austin, MN, USA) as previously described [40]. Mice were randomly assigned at 10 weeks of age to either a Low-Fat group fed commercial rodent diet (3.3 kcal/g) (*n *= 20) or to a High-Fat group fed a moderately high-fat diet consisting of 48% rodent diet, 44% condensed milk, and 8% corn oil (4.47 kcal/g) (*n *= 50). Mice were individually caged, and food intakes and body weights were determined weekly, at which time mice were palpated to identify the presence of MTs. Once MTs were detected, growth was monitored with calipers. Mice fed the high-fat diet with final body weights ± 2 standard deviations of Low-Fat mice were classified as Obesity-Resistant (*n *= 17). The remaining High-Fat mice were designated as Obesity-Prone (*n *= 33). Mice were euthanized when MT size reached 20 mm or at 85 weeks of age. When possible, blood samples were obtained by cardiac puncture before the mice were euthanized and serum prepared for leptin measurement. Serum leptin levels were determined using a commercial kit in accordance with the manufacturer's protocol (Linco Mouse Leptin Kit; Millipore Corporation). MTs and parametrial and retroperitoneal fat pads were removed and weighed. MFP samples were obtained but not weighed. MTs and tissue samples that appeared abnormal were preserved in formalin and sent to the Department of Pathology and Laboratory Medicine of the Mayo Foundation (Rochester, MN, USA) for histopathological analyses to determine malignancy and/or disease status. The remaining tissue was stored at -80°C until used. All procedures with mice were performed under the guidelines and with approval of the University of Minnesota Institutional Animal Care and Use Committee.

### Western blot analysis of mammary tumor and mammary fat pad proteins

Tissue samples were homogenized in extraction buffer with protease inhibitors. Total protein was extracted using a Total Protein Extraction Kit (Chemicon International, Temecula, CA, USA) and quantitated using a Bio-Rad protein assay kit with bovine serum albumin as a standard (Bio-Rad Laboratories, Inc.). Extracted proteins were electrophoresed on 4% to 15% polyacrylamide gradient gels and then transferred to a PVDF membrane. Blots were blocked in Tris-Base solution containing 1% milk concentrate and 0.15% Tween-20. The PVDF membranes were incubated with appropriate primary antibodies against leptin signaling proteins (leptin, long [ObRb] and total [ObR] forms of the leptin receptors, Jak2, pAkt, and pSTAT3) and apoptosis signaling proteins (Bcl-2, Bcl-xL, Bax, and PARP) in MFP and MT samples. YES Membranes were next incubated with a secondary antibody conjugated to alkaline phosphatase. ECF substrate was used to visualize the bands using a Storm 840 Machine Imaging System (Molecular Dynamics, Sunnyvale, CA, USA). Standard molecular weight markers were run simultaneously for comparing molecular weights of the visualized proteins. The intensity of Western blot bands was quantified by densitometric analysis using the program UN-SCAN-IT gel (Silk Scientific, Inc., Orem, UT, USA). Results were expressed as the ratio of intensity of the protein of interest to that of β-actin from the same sample. Although some mice had more than one MT, only one MT per animal was analyzed. For each measurement, 'n' is 3 to 6 for MT and 4 to 6 for MFP samples, as indicated in figure legends. Because no differences were found for MT and MFP samples from Obesity-Prone and Obesity-Resistant mice, these results were combined and designated as High-Fat.

### Measurement of caspase-3 activity in mammary tumor and mammary fat pad

Caspase-3 activity was determined as described in the manufacturer's protocol (R&D Systems, Inc., Minneapolis, MN, USA), using the same protein sample prepared for Western blot analysis. This assay is based on detection of cleavage of the substrate DEVD-AFC. The assay was carried out in 96-well flat-bottom microplates. Briefly, 100 μg of protein for MT and 150 μg of protein for MFP samples were incubated with 200 μM Ac-DEVD-pNA caspase-3 substrate in 50 μL of caspase assay buffer at 37°C for 4 hours. The release of pNA was measured at 400-nm excitation and 505-nm emission using a fluorometric Fluoroskan Ascent plate reader (Labsystem, now part of Thermo Fisher Scientific, Inc. Waltham, MA, USA). The fluorescence value obtained with the substrate, but without sample protein, was used as a background/negative control. Caspase-3 activity levels were calculated by subtracting the background value from the sample value. Each sample was run in duplicate. Values from the Low-Fat group were taken as 100%, and results from the High-Fat group were calculated relative to the Low-Fat group as a percentage.

### RT-PCR analysis of mRNA expression in mammary tumor and mammary fat pad samples

mRNA expression levels of leptin and long form (ObRb) and total form (ObR) of leptin receptors and STAT3 were measured in MT and MFP samples using reverse transcription-polymerase chain reaction (RT-PCR) analysis. Briefly, total RNA was extracted from equal amounts of frozen tissue samples using an RNeasy mini kit in accordance with the manufacturer's protocol (Qiagen Inc., Valencia, CA, USA). Isolated total RNA was quantified using a spectrophotometer, and equal amounts of RNA from each tissue were used for RT-PCR. Total RNA was reverse-transcribed using random hexamers, RNase inhibitor, reverse transcriptase (Superscript II), and oligo(dT) primers to synthesize first-strand cDNA from mRNA (Invitrogen Corporation). Total volume was adjusted to 50 μL using DNase- and RNase-free water. PCR primers were designed using nucleotide sequence for mouse ObR (GenBank accession number: U42467), ObRb (GenBank accession number: U58861), leptin (GenBank accession number: NM_008493), and STAT3 (GenBank accession number: U06922). The following primers were used: ObR, forward 5'-CAGATTCGATATGGCTTAAGT-3' and reverse 5'-GTTAAAATTCACAAGGGAAGC-3'; ObRb, forward 5'-ACACTGTTAATTTCACACCAGAG-3' and reverse 5'-TGGATAAACCCTTGCTCTTCA-3'; leptin, forward 5'-CCTGTGGCTTTGGTCCTATCTG-3' and reverse 5'-AGGCAGGCTGGTGAGGACCTG-3'; and STAT3, forward 5'-CAGGGTGTCAGATCACATGG-3' and reverse 5'-TTATTTCCAAACTGCATCAATG-3'.

These primers gave final product sizes of 475, 446, 244, and 600 base pairs (bp) for ObR, ObRb, leptin, and STAT3, respectively. PCR was performed under the following conditions: 94°C for 4 minutes for denaturing, 35 cycles of 94°C for 30 seconds, 62°C for 30 seconds, 72°C for 1 minute, and final extension at 72°C for 10 minutes. Reactions were carried out in a final volume of 25 μL in a thermocycler in the presence of Taq DNA polymerase. Water was used as the negative control, and mouse β-actin primers that produce a product of approximately 600 bp were used as internal controls. PCR products were separated on 1% agarose gel and stained with ethidium bromide, and product size was determined by concurrently separating 100-bp DNA ladder on the same gel. The DNA gel was scanned, the intensity of bands was quantified by densitometry using the program UN-SCAN-IT gel (Silk Scientific, Inc.), and results were expressed as the ratio of intensity of the gene of interest to that of β-actin in samples from individual mice. DNA obtained from tissue samples was run on agarose gel and subsequently extracted. The samples were sent to the Advanced Genetic Analysis Center of the University of Minnesota (St. Paul, MN, USA) for sequencing to confirm that the bands analyzed are the genes of interest (that is, ObR, ObRb, leptin, and STAT3).

### Statistical analysis

Results are presented as mean ± standard error of the mean (SEM). Tumor incidence data were analyzed by the chi-square test and Fisher exact test, and latency data were analyzed by the proportional hazard model using the Low-Fat group as the reference. Comparisons among three groups were made by analysis of variance (ANOVA) followed by the Newman-Keuls test to determine differences between specific groups. When only High-Fat versus Low-Fat groups were compared, the Student *t *test was used. The Pearson correlation test was used to determine whether the correlation between two parameters was significant.

## Results

### Caloric intake, body and fat pad weights, and serum leptin

**Figure 1:**
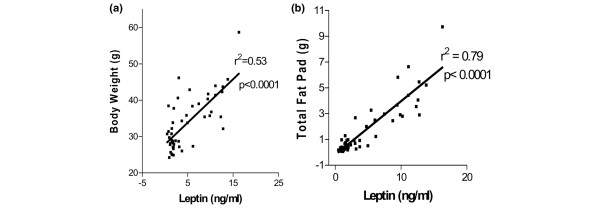
Correlation of serum leptin levels with fat pad and body weight in MMTV-TGF-α mice. **(a) **Correlation between serum leptin levels and total fat pad (*p *< 0.0001; *n *= 49). **(b) **Correlation between serum leptin levels and total body weight (*p *< 0.0001; *n *= 49).

There were no differences in caloric intake among the groups: Obesity-Prone, 9,481 ± 193 (*n *= 18); Obesity-Resistant, 8,647 ± 216 (*n *= 11); and Low-Fat, 9,157 ± 387 (*n *= 19) (values are mean ± SEM, ANOVA *p *= 0.219, calculated for mice that lived to the termination of the study). Obesity-Prone mice were 45% heavier than either Obesity-Resistant or Low-Fat mice at the termination of the experiment (*p *< 0.001) (Table 1). Additionally, combined retroperitoneal and parametrial fat pad weights of Obesity-Prone mice were 4.5 and 9 times heavier than those of Obesity-Resistant and Low-Fat mice, respectively (Table 1). Interestingly, fat pads from Obesity-Resistant mice were twice as heavy as those of Low-Fat mice despite the similar body weights of these two groups. However, the fat pad weights for Obesity-Resistant and Low-Fat mice were not significantly different by the Newman-Keuls test following ANOVA, when these values were analyzed by Student *t *test (*p *= 0.0174). Serum leptin levels were significantly higher in Obesity-Prone mice compared with Obesity-Resistant (*p *< 0.01) and Low-Fat (*p *< 0.001) mice (Table 1). Similar to fat pad weights, Obesity-Resistant mice had serum leptin levels that were twice those of Low-Fat diet mice; again, this difference was not significant using the Newman-Keuls test following ANOVA but was significantly different when compared by the Student *t *test (*p *< 0.03). When data from all mice were pooled, there was a significantly (*p *< 0.0001) positive correlation of fat pad and body weights to serum leptin levels (Figure 1a,b). Similar results were obtained for individual groups (data not shown).

**Table 1:** Body and fat pad weights and serum leptin for Obesity-Prone, Obesity-Resistant, and Low-Fat MMTV TGF-α mice

	Body weight (g)	Fat pad weight (g)	Serum leptin (ng/mL)
Obesity-Prone	40.49 ± 1.03^a,b ^(*n *= 33)	3.512 ± 0.41^b ^(*n *= 33)	10.56 ± 1.95^b ^(*n *= 25)
Obesity-Resistant	28.57 ± 0.51^c ^(*n *= 17)	0.786 ± 0.15^c ^(*n *= 17)	3.61 ± 1.11^c ^(*n *= 10)
Low-Fat	27.28 ± 0.56^c ^(*n *= 20)	0.389 ± 0.05^c ^(*n *= 20)	1.58 ± 0.15^c ^(*n *= 16)
Overall significance	*P *< 0.001	*P *< 0.001	*P *< 0.001

aValues are mean ± standard error of the mean. Different superscripts (b and c) indicate significant difference by Newman-Keuls test following analysis of variance.

### Mammary tumor development

Obesity-Prone mice had an overall MT incidence of 79% compared with 71% for Obesity-Resistant mice and 55% for Low-Fat mice (Table 2). Overall MT incidence is defined as (a) all MTs classified as adenocarcinomas and initially detected by palpation and (b) those nonpalpable MTs discovered at necropsy. These values were not significantly different from each other, although chi-square analysis between Obesity-Prone and Low-Fat mice had a *p *value of 0.07. When incidence rates for palpable MTs were determined, values were 55%, 29%, and 10% for Obesity-Prone, Obesity-Resistant, and Low-Fat mice, respectively. These results were significantly different for comparisons among the groups (*p *= 0.0038) with specific differences between Obesity-Prone and Obesity-Resistant (*p *= 0.09), Obesity-Prone and Low-Fat (*p *= 0.0012), and Obesity-Resistant and Low-Fat (*p *= 0.21) (Table 2).

**Table 2:** Mammary tumor incidence, latency, and weight and number for MMTV TGF-α mice

	Overall mammary tumor incidence (percentage)	Palpable mammary tumor incidence (percentage)	Palpable mammary tumor latency, weeks	Mammary tumor weight, grams^a^	Mammary tumor number^a^
Obesity-Prone	26/30 (79)^b,c^	18/33 (55)^d^	74.1 ± 2.4^e^	2.407 ± 0.32^f^	3.9 ± 0.08
Obesity-Resistant	12/17 (71)	5/29 (29)	76.7 ± 2.1	1.298 ± 0.35^g^	3.5 ± 0.20
Low-Fat	11/20 (55)	2/20 (10)	81.9 ± 0.1	0.653 ± 0.29^g^	2.3 ± 0.18

^a^Values from tumor-bearing mice only: Obesity-Prone (*n *= 26), Obesity-Resistant (*n *= 12), and Low-Fat (*n *= 11). Analysis of variance for statistical comparison.

^b^Chi-square *p *= 0.07 among the groups; Obesity-Prone versus Low-Fat, *p *= 0.06.

^c^Low-grade adenocarcinomas confirmed by histopathology except for six high-grade adenocarcinomas in six different Obesity-Prone mice.

^d^Chi-square *p *= 0.0038 among the groups; Obesity-Prone versus Low-Fat, *p *= 0.0012; Obesity-Prone versus Obesity-Resistant, *p *= 0.09; Obesity-Resistant versus Low-Fat, *p *= 0.21.

^e^Differences among groups significant by log-rank test, *p *= 0.0015. This was followed by proportional hazard model: Obesity-Prone versus Low-Fat, *p *= 0.0041, and Obesity-Resistant versus Low-Fat, *p *= not significant.

Different superscripts (f and g) indicate significant difference.

There was a significant difference among the groups with respect to age of palpable MT detection (Table 2). There was also a significant difference between the Low-Fat and Obesity-Prone groups of almost 8 weeks. The latency for overall MT development is not shown as the results are affected by the fact that most of the MTs in the Low-Fat and Obesity-Resistant groups were found at 85 weeks of age, when the mice were euthanized because this was the termination point of the study.

Average MT weights per tumor-bearing mouse were 2.41, 1.30, and 0.65 g for Obesity-Prone, Obesity-Resistant, and Low-Fat mice (*p *= 0.0016), respectively (Table 2). Although the average MT weight for Obesity-Resistant mice was twice that of Low-Fat mice, this did not reach statistical significance. There was a trend toward increased MT number per tumor-bearing mouse for mice fed the high-fat diet regardless of body weight classification; however, this did not reach statistical significance either. Interestingly, six Obesity-Prone mice had MTs classified as high-grade adenocarcinoma compared with none for the other two groups.

### Leptin signaling protein and mRNA expression in mammary tumor and mammary fat pad

**Figure 2:**
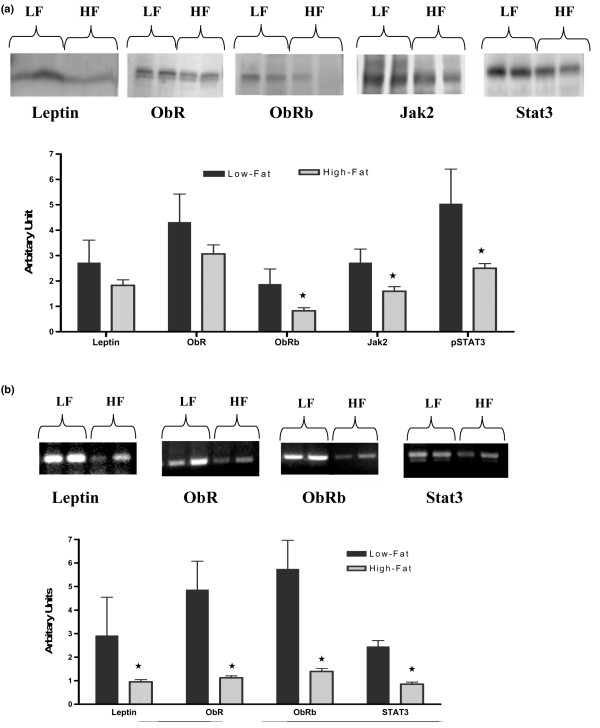
Densitometric analysis of leptin signaling protein and mRNA expression levels in mammary tumor (MT) of Low-Fat and High-Fat mice. **(a) **Leptin signaling protein expression in MT. **(b) **Leeptin mRNA expression levels in MT. Protein bands were obtained using Western blot analysis as described in Materials and methods. Results are the average of three and six MT samples of individual mice. *p < 0.05. HF, High-Fat; Jak2, Janus kinase 2; LF, Low-Fat; pSTAT3, phosphorylated signal transducer and activator of transcription 3; STAT3, signal transducer and activator of transcription 3.

Because there were no significant differences between Obesity-Prone and Obesity-Resistant mice, protein expression levels for these two groups were combined and termed High-Fat. Protein expression levels of ObRb, Jak2, and pSTAT3 were significantly lower in MT samples from High-Fat mice compared with those from Low-Fat mice (*p *< 0.05) (Figure 2a). On the other hand, leptin and ObR protein expression levels were similar between the two groups. In addition, MTs obtained from Low-Fat mice had significantly higher mRNA expression levels of leptin, ObR, ObRb, and STAT3 (*p *< 0.01) than those of High-Fat mice (Figure 2b).

**Figure 3:**
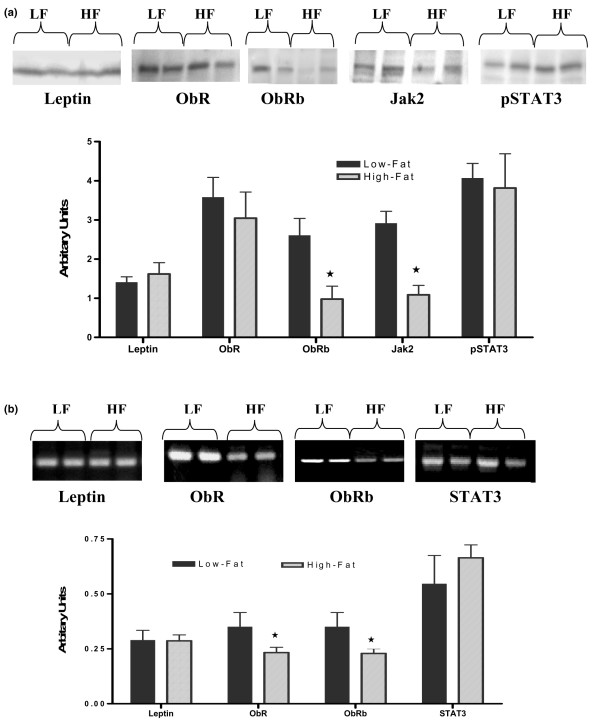
Densitometric analysis of leptin signaling protein and mRNA expression levels in mammary fat pad (MFP) from Low-Fat or High-Fat mice. **(a) **Leptin signaling proteins in MFP. Protein bands were obtained using Western blot analysis as described in Materials and methods. Data are from five Low-Fat and four High-Fat mice. **(b) **Average densitometry of leptin signaling molecule mRNA expression levels in MFP. Average density values of the mRNA expression bands were from five Low-Fat and six High-Fat mice. *p < 0.05. HF, High-Fat; Jak2, Janus kinase 2; LF, Low-Fat; pSTAT3, phosphorylated signal transducer and activator of transcription 3; STAT3, signal transducer and activator of transcription 3.

MFP samples from High-Fat mice exhibited significantly lower protein levels of ObRb and Jak2 compared with MFP from Low-Fat mice (*p *< 0.05) (Figure 3a). However, there were no significant differences between MFP samples from Low-Fat and High-Fat mice for leptin, ObR, and pSTAT3 protein levels. Expressions of ObR and ObRb mRNA levels were significantly higher in MFP of Low-Fat compared with High-Fat mice (Figure 3b). However, leptin and STAT3 mRNA expression levels were similar in MFP samples from both groups (Figure 3b).

### Apoptosis signaling proteins in mammary tumor and mammary fat pad

**Figure 4:**
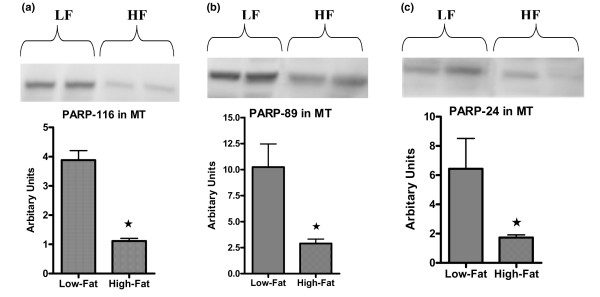
Western blot analysis for poly(ADP-ribose)polymerase (PARP) protein expression in mammary tumor (MT). Total **(a) **and cleaved PARP **(b, c) **protein expression levels were analyzed using Western blot analysis as described in Materials and methods. Data are average density values of three and six individual mice from Low-Fat (LF) and High-Fat (HF) groups, respectively. *p < 0.05.

**Figure 5:**
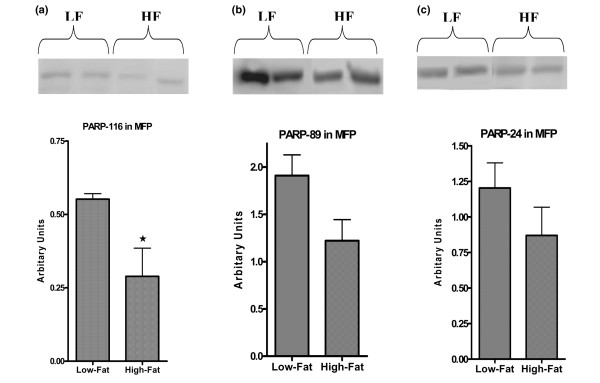
Western blot analysis of poly(ADP-ribose)polymerase (PARP) protein expression in mammary fat pad (MFP). Total **(a) **and cleaved PARP **(b, c) **protein expression levels were analyzed using Western blot analysis as described in Materials and methods. Data are average density values of five and four individual mice from Low-Fat (LF) and High-Fat (HF) groups, respectively. *p < 0.05.

To investigate the effects of body weight and diet on the apoptosis signaling pathway, a number of protein expression levels were determined. Total PARP (116 kDa) and two of its cleaved products (89 and 24 kDa) in MT and MFP tissues were measured. Low-Fat mice had significantly higher (3.5-fold) levels of total and cleaved PARP products in MTs compared with High-Fat mice (*p *< 0.01) (Figure 4a–c). In MFP of Low-Fat mice, the total PARP expression level was significantly higher than that of High-Fat mice (*p *< 0.01) (Figure 5a). On the other hand, there were no significant differences between the two groups for cleaved PARP products (Figure 5b,c), although there was a trend for the 89-kDa product of PARP (*p *= 0.08) to be higher in MFP obtained from Low-Fat mice (Figure 5b).

**Figure 6:**
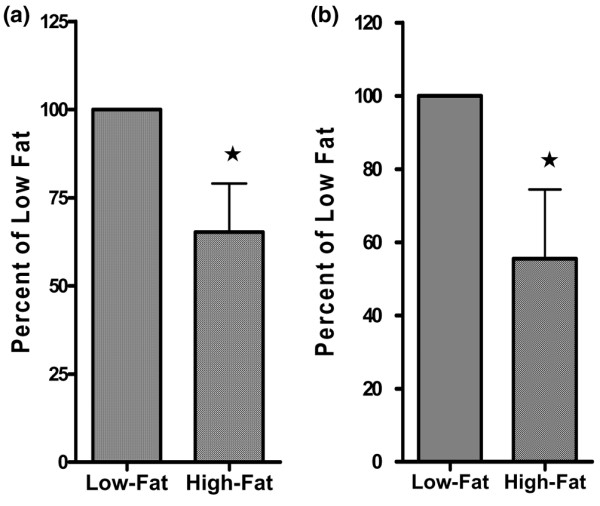
Levels of caspase-3 activity in mammary tumor (MT) and mammary fat pad (MFP). **(a) **Caspase-3 activity in MT from three Low-Fat and six High-Fat mice. **(b) **Caspase-3 activity in MFP from five Low-Fat and four High-Fat mice. *p < 0.05.

The mitochondrial apoptosis pathway was also assessed by determining the activity of caspase-3, which cleaves PARP. Activity of caspase-3 was significantly higher, by 35%, in MTs from Low-Fat versus High-Fat mice (*p *< 0.05) (Figure 6a,b). Similarly, activity of caspase-3 in MFP obtained from Low-Fat mice was 45% higher than that of High-Fat mice (*p *< 0.05) (Figure 6b).

**Figure 7:**
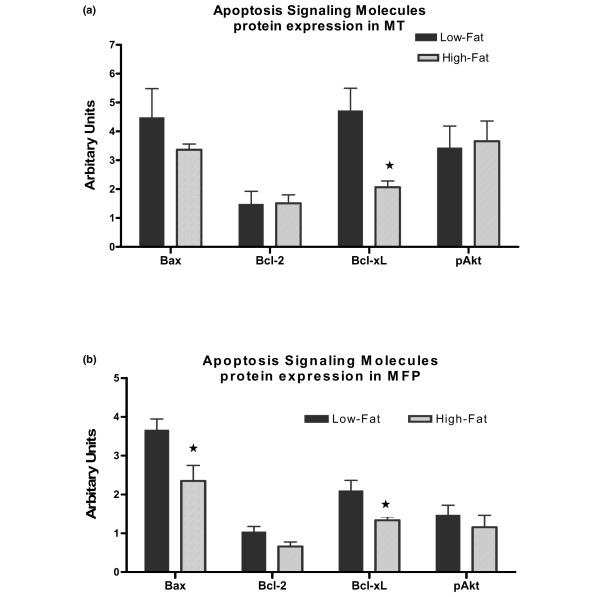
Densitometric analysis of apoptosis signaling pathway protein expression in mammary tumor (MT) and mammary fat pad (MFP) from MMTV-TGF-α mice. **(a) **MT samples obtained from three Low-Fat and six High-Fat mice. **(b) **MFP samples obtained from five Low-Fat and four High-Fat mice. *p < 0.05. pAkt, phosphorylated Akt.

Levels of Bcl-xL protein expression were significantly (*p *< 0.05) higher in MTs from Low-Fat mice compared with this protein in MTs from High-Fat mice (Figure 7a). The expression levels of Bax, Bcl-2, and pAkt proteins were similar in MT from both groups (Figure 7a). In MFP, protein expressions of Bax and Bcl-xL were significantly higher in Low-Fat mice (*p *< 0.05) (Figure 7b). However, there were no differences in protein expression levels of Bcl-2 and pAkt in MFP samples obtained from the mice fed either low-fat or high-fat diets (Figure 7b).

## Discussion

In the present study, we demonstrate that mice fed a high-fat diet from 10 to 85 weeks of age and designated Obesity-Prone had significantly higher body and fat pad weights and higher serum leptin levels in comparison with mice fed the same high-fat diet and designated as Obesity-Resistant or Low-Fat. In addition, MT incidence and MT weights were higher in Obesity-Prone mice compared with both Obesity-Resistant and Low-Fat mice, and palpable MT latency was shortened in Obesity-Prone mice compared with Low-Fat mice. Interestingly, there were also differences in comparisons between the Low-Fat and Obesity-Resistant mice despite their similar body weights, such as a trend toward higher palpable MT incidence and higher fat pad weights and serum leptin levels in the Obesity-Resistant mice.

The findings of higher MT weights and presence of high-grade adenocarcinomas in the Obesity-Prone mice indicate similarities to the relationship of obesity with human breast cancer. For example, obesity has been associated with bigger tumors, more advanced disease, poorer prognosis, and/or increased mortality in women with breast cancer [6]. The shortened MT latency reported for Obesity-Prone mice is consistent with human studies relating higher body weight/body mass index with increased risk of breast cancer. For example, the presence of obesity would promote the development of breast cancer sooner in susceptible women, who if they had stayed lean might eventually die of causes other than breast cancer. Also, if breast cancer did develop at a later age, its consequences may have less impact on shortening life expectancy and/or affecting quality of life. Furthermore, our results suggest that body fat independent of body weight and obesity may be an important factor in MT development as indicated by differences between Obesity-Resistant and Low-Fat mice. This was seen by the many intermediate measurements for the Obesity-Resistant mice and by the fact that gene expression data were impacted more by consumption of the high-fat diet that increased body fat levels.

A major focus of the present study was to determine the impact of diet and/or body weight on expression of leptin signaling proteins in MFP and MT tissues. This was of interest as a consequence of earlier studies indicating that the absence of leptin and its receptor were associated with a lack of MT development in transgenic MMTV-TGF-α mice [27,29]. The fact that the leptin receptor was identified in human breast tumors and in human breast cancer cell lines and that the addition of leptin to breast cancer cell lines increased cell proliferation [17-19,41-43] further supported the involvement of leptin in mammary tumorigenesis. Also, while this long-term mouse experiment was under way, additional publications further supported that leptin stimulates proliferation and affects signal transduction in breast cancer cell lines [41-45].

Despite the increasing evidence that leptin impacts human breast cancer cells *in vitro*, the role of leptin in the development of human breast cancer *per se *is not clear. Some studies have reported that breast cancer subjects have higher serum leptin levels than controls, but others have not [46-50]. Since some of these studies focused on younger subjects or combined pre- and postmenopausal women and subject numbers were small, it is not surprising that inconclusive results have been obtained so far. Additionally, as in the present study, serum was obtained at only one time point, usually at diagnosis. Several recent studies have suggested that serum leptin levels in association with expression of leptin and/or leptin receptor isoforms in breast tumors or with gene polymorphisms for leptin and its receptor may be more definitive in identifying the impact of serum leptin on prognosis with respect to disease recurrence and death [51,52]. Although as indicated leptin and leptin receptor expression have been identified in human breast tumors, proteins associated with leptin signaling have not been characterized. In the present study, we not only found that leptin receptors ObR and ObRb are present in MTs obtained from MMTV-TGF-α mice, but we detected the presence of Jak2 and pSTAT3 proteins, which are located in the downstream pathway of leptin signaling. These proteins were also shown to be expressed in MFP. Additionally, the presence of leptin itself was detected in both MFP and MT samples, indicating the potential for paracrine and/or autocrine action of leptin. Furthermore, we found that consumption of a high-fat diet was associated with lower protein expression levels of ObRb and Jak2 in MFP and with lower ObRb, Jak2, and pSTAT3 protein expression levels in MT samples.

Our findings of a reduction in ObRb and/or Jak2 and pSTAT3 protein expression levels are similar to previous reports for effects of diet-induced obesity and consumption of high-fat diets on these proteins in the hypothalamus. For example, in male (Fischer 344 × Brown Norway) rats with diet-induced obesity as a consequence of consumption of a high-fat diet, ObRb protein and mRNA expressions were reported to be reduced in the hypothalamus compared with chow controls [53]. In addition, 30 days of calorie restriction after the 105 days of consuming the high-fat diet resulted in increased ObRb and pSTAT3 protein expression in diet-induced obese rats. In other studies, the reduction of leptin receptor expression levels in the hypothalamus of high-fat-fed Osborne-Mendel rats [54] and C57BL6 mice [55] in comparison with low-fat counterparts also parallels our findings in MFP and MT tissues. In adipose tissue from male C57BL6 mice fed a high-fat diet for 8 weeks, higher serum leptin levels were associated with reduced expression levels of the short form of the leptin receptor, although expression levels of the long form of the leptin receptor were similar to those of low-fat control mice [56]. This reduction in the leptin receptor and/or components of the signaling pathway has been termed leptin resistance and is associated with genetic and diet-induced obesities as well as with aging [57-60].

In the present study, we also assessed effects of low-fat versus high-fat diets on expression levels of apoptosis-related proteins in MTs and MFP. All of the proteins, total PARP, PARP-89, PARP-24, Bax, Bcl-2, Bcl-xL, and caspase-3 activity, were detected in both tissues. Furthermore, we found that diet affected most of these proteins more than body weight status, with the consumption of a high-fat diet being associated with reduced apoptosis in both MTs and MFP. Leptin has been reported to inhibit apoptosis in human colon [37] and prostate [38] cancer cell lines and in leukemic cells [39]. Since mice fed the high-fat diet had higher serum leptin levels compared with those fed the low-fat diet, the lower expression of these apoptotic proteins is consistent with *in vitro *results. We also found that the consumption of a high-fat diet resulted in significantly decreased cleaved PARP protein expression levels in MT and MFP samples. In addition, the activity of caspase-3, which cleaves total PARP, was lower in MFP and MT tissues of the mice fed the high-fat diet. Both caspase-3 and cleaved PARP products are involved in DNA breakage and lead to apoptosis. Although in the present study cell survival protein expression levels either did not change (pAkt and Bcl-2) or were decreased (Bcl-XL) in high-fat diet mice that also had higher serum leptin levels, in an *in vitro *study with HTB-26 and ZR75-1 breast cancer cell lines, it was shown that leptin increased the expression levels of pERK (phosphorylated extracellular signal-regulated kinase) and pAkt [41]. This difference may well be a consequence of the different study designs (*in vitro *versus *in vivo*). In particular, in the present study, tissues were removed after long-term diet maintenance and exposed to many other hormones and cytokines in addition to leptin in the body, whereas in the other study, cells were maintained with leptin for only 48 hours after serum deprivation for 20 hours *in vitro*.

A summary of the results of protein and mRNA expression levels is presented in Table 3. The effects of high-fat diet relative to low-fat diet were similar on the expression levels of leptin, ObR, ObRb, Jak2, total PARP, caspase-3, Bcl-2, Bcl-Xl, and pAkt protein both in MT and MFP tissues. Overall changes that did occur were primarily in the direction of reduced apoptosis in the MFP and MT tissues obtained from mice fed the high-fat diet. The present study is the first to show that consumption of a high-fat diet (for 75 weeks) has effects on pro-apoptotic and survival protein expression levels in MTs and MFP. However, several previous publications have studied the effects of calorie restriction on similar protein expression levels. For example, levels of pro-apoptotic Bax, caspase-3 activity, and cleaved PARP products were all higher in MTs from energy-restricted rats (40% reduction) compared with MTs from *ad libitum*-fed rats, whereas Bcl-2 and pAkt were reduced [61]. In another study, using MTs obtained from energy-restricted MMTV-TGF-α mice, caspase-3 activity and DNA breakage were elevated in mice subjected to 50% restriction but not to 25%, but pAkt was not affected [62]. In the present study, consumption of a high-fat diet resulted in little change on cell survival proteins (Bcl-2, Bcl-xL, and pAkt) in MT as well as MFP tissues, with the primary effect of consumption of the high-fat diet resulting in reduced apoptosis. Thus, calorie restriction appears to promote apoptosis in MTs whereas consumption of the moderately high-fat diet, even without effects on caloric intake, did not.

**Table 3:** Summary of diet effects on leptin and apoptosis signaling protein and mRNA expression levels

	MT		MFP	
Leptin signaling molecules				
Leptin	NC		NC	NC
ObR	NC		NC	
ObRb				
Jak2		NM		NM
pSTAT3			NC	NC
Apoptosis signaling molecules				
PARP (total)				
PARP (89 kDa)			NC	
PARP (24 kDa)			NC	
Caspase-3 activity				
Bax	NC			
Bcl-2	NC		NC	
Bcl-XL				
pAkt	NC		NC	

Jak2, Janus kinase 2; MFP, mammary fat pad; MT, mammary tumor; NC, no statistically significant change; NM, not measured; pAkt, phosphorylated Akt; PARP, poly(ADP-ribose)polymerase; pSTAT3, phosphorylated signal transducer and activator of transcription 3. Solid arrows show protein expression and dashed lines mRNA expression.

Although it is documented that consumption of high-fat diets shortens MT latency, the molecular mechanism mediating this effect is unknown. In the present study, we report that mice that were fed a high-fat diet and that became obese had higher MT incidence and serum leptin levels, which were correlated with total fat pad and body weights. We also demonstrated that expression levels of leptin and apoptotic-associated proteins were modulated by long-term high-fat diet consumption. Leptin resistance appears to be associated with high-fat diet consumption in both MTs and MFP, and an anti-apoptotic protein profile characterizes the MTs from mice fed the high-fat diet. At this point, it is not clear what would happen at time points prior to the development of leptin resistance and how this would relate to MT development.

## Conclusion

These results indicate that dietary-induced obesity enhances MT development in MMTV-TGF-α mice compared with mice that were fed the same high-fat diet and that did not become obese as well as with mice that were fed a low-fat diet. However, the expressions of a number of proteins associated with leptin and leptin signaling in both the MTs that formed and in MFP tissues were primarily affected by diet. This may be a result of the long-term consumption of the high-fat diet leading to leptin resistance. Future studies will be directed toward shorter-term diet intervention and specific effects on mammary tissues, particularly epithelial cells, to try to identify mechanistic explanations for how body fat could impact MT development. More studies need to be done to clarify how other signaling proteins and pathways are affected by low-fat versus high-fat diets as well as by caloric restriction and body weight/body fat changes, so that pharmacological drugs can be developed to target those molecules.

## Abbreviations

ANOVA = analysis of variance; bp = base pairs; ECF = enhanced chemifluorescence; IgG = immunoglobulin G; Jak2 = Janus kinase 2; MFP = mammary fat pad; MT = mammary tumor; pAkt = phosphorylated Akt; PARP = poly(ADP-ribose)polymerase; pSTAT3 = phosphorylated signal transducer and activator of transcription 3; PVDF = polyvinylidene difluoride; RT-PCR = reverse transcription-polymerase chain reaction; SEM = standard error of the mean; STAT3 = signal transducer and activator of transcription 3.

## Competing interests

The authors declare that they have no competing interests.

## Authors' contributions

MPC is the principal investigator of the present study and contributed to the analysis and interpretation of the data and drafting of the manuscript. SD contributed to the analysis and interpretation of the data and drafting of the manuscript. XH contributed to animal dissection and tissue processing. YZ performed statistical analysis. NJM provided assistance in data interpretation and manuscript preparation. JPG read the pathological slides from the tissues taken from the animals to decide whether the tissues were tumors or not, in addition to classifying tumor grades. All authors contributed to revisions of the manuscript and approved the submitted manuscript.
